# The Effects of Physical Exercise on the Quality of Life of Healthy Older Adults in China: A Systematic Review

**DOI:** 10.3389/fpsyg.2022.895373

**Published:** 2022-06-20

**Authors:** Lin Wei, Yongmei Hu, Yingying Tao, Rui Hu, Liancheng Zhang

**Affiliations:** Key Laboratory of Competitive Sport Psychological and Physiological Regulation, Tianjin University of Sport, Tianjin, China

**Keywords:** physical exercise, quality of life, older adults, systematic review, investigative studies, experimental studies

## Abstract

**Objective:**

To systematically evaluate the effects of physical exercise on the quality of life (QOL) of healthy older adults in China.

**Methods:**

Relevant articles published until December 2021 were retrieved from China National Knowledge Infrastructure, Wanfang, China Science and Technology Journal Database, PubMed, EBSCO, Web of Science, and the Library of Congress. Inclusion criteria were studies in which the subjects were healthy Chinese older adults (aged ≥ 60 years), the reported sample size was clear, and the study design was a randomized controlled trial or a research study. In addition, studies were included if they reported the use of at least one QOL questionnaire and investigated at least one form of physical exercise.

**Results:**

In total, 19 studies met the inclusion criteria, which included six studies that used comprehensive physical exercise type as an intervention and 13 studies that used regular physical exercise as an intervention. All 19 studies compared intervention and control groups, of which 12 (63%) were investigative studies and seven (37%) were experimental studies. Of the experimental studies, five used a positive control group and two used a negative control group. All 19 studies reported that physical exercise had varying degrees of positive effects on the QOL in older adults. Body–mind exercise was effective in improving the physical and mental health (MH) of older adults, whereas Xiyangcao only had a positive effect on physical health. Compared with no exercise or other exercise (exercise not used in the intervention group), the exercise group in the survey had a positive effect on the QOL of older adults. Regardless of the type of control group used, the exercise group in the experimental studies showed a positive effect of exercise on the QOL of older adults.

**Conclusion:**

Physical exercise has a positive impact on the QOL of healthy older adults. However, due to the wide and varied scope of the included studies, more randomized controlled trials are needed to examine the effects of different types, intensities, durations, and the frequency of exercise on QOL.

**Systematic Review Registration:**

[https://www.crd.york.ac.uk/prospero/display_record.php?RecordID=220115], identifier: [CRD42020220115].

## Introduction

Quality of life (QOL) was first put forward by the US economist J. Calbraith in the 1950s (Wright and Doughty, 1993). Subsequently, the World Health Organization (WHO) defined it as the experience of individuals in different cultures and value systems about their life goals, expectations, standards, and living conditions of the things they care about (The Whoqol Group, 1994). Recently, the growing aging population has called for additional attention to be paid to the QOL of older adults in the society. The QOL of older adults is an important indicator of healthy aging (Fu and Xie, 2010), a comprehensive indicator of good physical and mental states (MS) (Li and He, 2002), and an accurate indicator of overall health status (Zhu et al., 2009).

Previous studies have shown that physical exercise is one of the main factors affecting QOL in older adults (Fei and Liu, 2016). However, among the eight meta-analyses conducted on the impact of exercise on QOL, five only analyzed within groups, with no comparisons to the control group, three reported no beneficial effect of physical exercise on QOL (Schmitz et al., 2005; Spronk et al., 2005; Jolly et al., 2006), and two reported positive effects of exercise on QOL (Oldervoll et al., 2004; Van Tol et al., 2006). The remaining three studies compared exercise and control groups, of which one study reported no effect (Lawlor and Hopker, 2001), and the other two reported positive effects of exercise (Schechtman et al., 2001; Netz et al., 2005). These discrepancies in findings indicate the need to systematically evaluate existing studies on the basis of controlling the quality of studies and to comprehensively examine the impact of physical exercise on QOL in older adults. This will provide a basis for the subsequent formulation of physical exercise plans to help improve QOL in older adults.

Previous studies have investigated QOL in older adults; however, most studies have focused on non-healthy older populations, such as frail older adults (Campbell et al., 2021). Furthermore, little is known about the QOL of Chinese older adults, and the types of physical exercise that older adults engage in also vary from country to country. Therefore, it is necessary to explore the impact of physical exercise on QOL in healthy Chinese older adults.

The present systematic review included randomized control trials and studies in healthy older adults in China to summarize the types of physical exercise, exercise frequency, exercise duration, types of study design, and measurement tools. We conducted meta-integration according to the results of each QOL scale and its subdimensions [i.e., physical function (PF), role physical (RP), bodily pain (BP), general health (GH), vitality (VT), social function (SF), role emotional (RE), mental health (MH), physical component summary (PCS), mental component summary (MCS), material life (ML), MS, general state (GS), mental function (MF), physiological domain (PD), mental domain (MD), social domain (SD), environmental domain (ED), independent domain (ID), spiritual pillar (SP), and social relations (SR)]. This study aimed to contribute to this growing area of research by comprehensively and objectively exploring the impact of physical exercise on QOL in healthy Chinese older adults. In addition, the types of study design may be integrated to improve the value of physical exercise projects, provide a reference for the scientific rigor of future studies, and improve the QOL of older adults.

## Methods

This systematic review was conducted according to the Preferred Reporting Item for Systematic Review and Meta-Analyses (PRIMA) guidelines (Vrabel, 2009). The protocol of this systematic review was registered in PROSPERO (ID protocol: CRD42020220115).

### Search Strategy

Two reviewers (LW and YT) independently searched the China National Knowledge Infrastructure, Wanfang, and the China Science and Technology Journal Database using the Chinese terms of “physical exercise” OR “sports” OR “physical activity” OR “exercise” AND “older adults” AND “quality of life” in the article title, abstract, or keywords for articles published up to December 2021. In addition, the reviewers searched PubMed, EBSCO, Web of Science, and the Library of Congress databases using the English terms “physical exercise” OR “sports” OR “physical activity” OR “exercise” AND “quality of life” AND “Chinese older adults” OR “Chinese elderly” OR “the aged” OR “the old” in the article title, abstract, or keywords in articles published up to December 2021. The electronic search was supplemented by a manual search of the reference lists of the included review articles to identify any additional sources.

### Selection Criteria

The selection criteria to define the characteristics of the included studies were based on the PICOS criteria:

1. *Population*: studies that included healthy Chinese older adult participants aged 60 years or above were included (In China, the concept of older adults is officially defined as people aged 60 years and above. Therefore, we chose the study that included participants above 60 years).
2. *Intervention*: studies evaluating the effect of physical exercise were included.
3. *Comparator*: studies comparing other exercise types (e.g., walking) or no exercise were included.
4. *Outcomes*: studies that assessed QOL and its dimensions were included.
5. *Study design*: studies of any type (i.e., investigative studies or experimental studies) using any design (i.e., within-subject and between-subject designs) were included.

Two reviewers (LW and YT) initially performed the search for articles, after which the titles and abstracts of identified studies were screened for potential selection. Any disagreements were discussed with a third reviewer (RH) until a consensus was reached.

### Quality Assessment

We used the PEDRO scale, an 11-item quality assessment tool, to assess of the risk of bias. It combines two scales based on the Delphi table (Verhagen et al., 1998). The 11 items of the scale are described as follows: (1) eligibility criteria were specified (no points were awarded for this criterion); (2) subjects were randomly allocated to groups; (3) allocation was concealed; (4) the most important prognostic indicator was similar across all groups at baseline; (5) all subjects were blinded; (6) all therapists who administered the therapy were blinded; (7) all assessors who measured at least one key outcome were blinded; (8) the measurement of at least one key outcome was obtained for more than 85% of the subjects initially allocated to a group; (9) all subjects for whom outcome measures were available received the treatment or control condition as allocated or, where this was not the case, data for at least one key outcome were analyzed by “intention to treat”; (10) between-group statistical comparisons were reported for at least one key outcome; and (11) both point measures and measures of variability are provided for at least one key outcome. When a certain criterion was clearly met, 1 point was given to the study, whereas non-compliance was recorded as 0 point. The total scale score was 10 points, where <4 points indicated poor quality, 4–5 points indicated medium quality, 6–8 points indicated good quality, and 9–10 points indicated high quality. The methodological quality of each study was evaluated by two reviewers (LW and YT), and disagreements were resolved by consulting a third reviewer (RH).

### Data Extraction

Information on study details (i.e., author, year, sample size, average, standard deviation, types of physical exercise, exercise frequency, exercise duration, study design, measurement tools, and outcome variables) was extracted from all included studies. If there was any missing or vague information, the reviewers contacted the original author to obtain the corresponding information. Data from the included studies were extracted independently by two reviewers (LW and YT), and any discrepancies were solved by consulting a third reviewer (RH).

### Data Analysis

Due to the considerable variability of the research methods, a meta-analysis was not possible. Thus, a systematic review was conducted.

## Results

### Search Results

**FIGURE 1:**
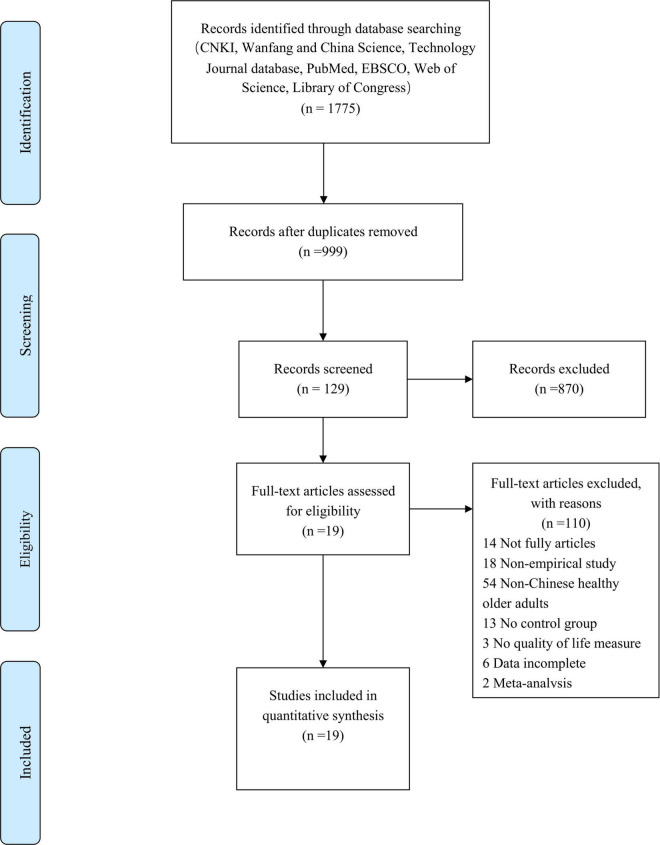
Article selection process.

A total of 1,775 articles were initially retrieved by searching for potential inclusion. After removing duplicates, 614 articles remained for further screening of titles and abstracts. Then, 129 full-text articles were assessed for examination of their eligibility. Finally, 19 articles were included in a systematic review (Figure 1).

### Quality of Studies

The quality of the 19 included articles was assessed according to the PEDRO scale. The scores ranged from 4 to 7 points (Table 1). None of the included studies used blinding of subjects or evaluators, which resulted in lower scores.

**TABLE 1:** PEDRO scores of the included studies.

Study	Evaluation criterion											Scores
	1	2	3	4	5	6	7	8	9	10	11	
Li et al., 2007	1	1	1	0	0	0	0	1	1	1	0	5
Li et al., 2016	1	1	1	0	0	0	0	1	1	1	0	5
Cui, 2014	0	0	1	0	0	0	0	1	1	1	0	4
Cu et al., 2002	1	0	1	0	0	0	0	1	1	1	0	4
Guo, 2019	1	1	1	0	0	0	0	1	1	1	0	5
You, 2009	1	1	1	1	0	0	0	1	1	1	0	6
Yang et al., 2013	0	0	1	0	0	0	0	1	1	1	0	4
Liao, 2005	1	0	1	0	0	0	0	1	1	1	0	4
Li et al., 2005	1	1	1	1	0	0	0	1	1	1	0	6
Chen and Liu, 2015	1	0	1	0	0	0	0	1	1	1	0	4
Wen, 2014	1	0	1	0	0	0	0	1	1	1	0	4
Wang, 2019	1	1	1	1	0	0	0	1	1	1	0	6
Liu et al., 2014	1	1	1	1	0	0	0	1	1	1	0	6
Jiang et al., 2009	1	1	1	0	0	0	0	1	1	1	0	5
Guan, 2017	1	1	1	1	0	0	0	1	1	1	0	6
Luo et al., 2008	1	1	1	0	0	0	0	1	1	1	0	5
Wang and Wang, 2020	0	1	1	1	0	0	0	1	1	1	0	6
Peng, 2009	0	0	1	1	0	0	0	1	1	1	0	5
Lee et al., 2010	1	1	1	1	0	1	0	1	1	1	0	7

*1. Eligibility criteria; 2. Randomization; 3. Allocation hidden; 4. Similar group baselines; 5. Blinding of all subjects; 6. Blinding of all therapists; 7. Blinding of all assessors; 8. Drop out ≤ 15%; 9. Intention to treat method; 10. Statistical comparisons between-groups; 11. Point measures and measures of variability.*

### Study Characteristics

All included studies addressed the effects of physical exercise on the QOL of healthy older adults in China. An overview of the included studies is provided in Table 2. The publication year ranged from 2002 to 2020, and the total sample size was 5,732. In total, 12 were investigative studies (63%) and seven were experimental studies (37%).

**TABLE 2:** Characteristics of studies included in the systematic review.

Study	Sample size		Types of physical exercise		Exercise frequency	Exercise duration	Study design	Measurement tools	Outcome variables
	I	C	I	C					
Li et al., 2007	158	172	Doing exercise in the morning	No exercise	More than four times per week	–	Survey	WHOQOL-100	PD, MD, ID, SP, SR, T
Li et al., 2016	374	108	Sporting activities at leisure time	No exercise	More than four times per week	More than 30 min	Survey	WHOQOL-BREF	PD, MD, SD, ED, T
Cui, 2014	379	146	Light physical exercise	Non-physical exercise	–	–	Survey	WHOQOL-BREF	PD, MD, SD, ED, T
Cu et al., 2002	170	571	Doing exercise in the morning	No exercise	–	–	Survey	WHOQOL-BREF	PD, MD, SD, ED
Guo, 2019	352	121	Doing a wide variety of exercise	No exercise	More than two times per week	More than 30 min	Survey	SF-36	PF, RP, BP, SF, RE, T
You, 2009	31	31	Liuzijue	No exercise	More than four times per week	60 min	Experiment	SF-36	RP, BP, GH, VT, SF, RE, MH
Yang et al., 2013	563	382	Doing exercise conducted at least three times per week	No exercise	More than five times per week	More than 30 min	Survey	SF-36	PF, RP, BP, GH, VT, SF, RE, MH, PCS, MCS
Liao, 2005	165	53	Doing exercise conducted at least three times per week	No exercise	–	–	Survey	SF-36	PF, RP, BP, GH, VT, SF, RE, MH, T
Li et al., 2005	118	42	Doing a wide variety of exercise	No exercise	More than three times per week	More than 30 min	Survey	SF-36	PF, BP, GH, VT, RE, MH
Chen and Liu, 2015	285	178	Doing exercise conducted at least three times per week	Doing exercise conducted less than three times per week	More than three times per week	More than 30 min	Survey	SF-36	PF, RP, GH, VT, SF, RE, MH, T
Wen, 2014	317	222	Sporting activities at leisure time	No exercise	–	–	Survey	SF-36	PF, RE, VT, GH, PCS, MCS
Wang, 2019	18	17	Xiyangcao	Walk slowly	Three times per week	60 min	Experiment	SF-36	PF, BP, PCS
Liu et al., 2014	47	48	Baduanjin	Walk	Two times per week	30–40 min	Experiment	SF-36	BP, GH, VT, SF, RE, MH, T
Jiang et al., 2009	41	46	High-intensity exercise	Lack of exercise	More than three times per week	More than 30 min	Survey	SF-36	PF, BP, GH, VT, SF, RE, MH, T
Guan, 2017	30	30	New exercise prescription	Tai Chi, Square dancing	Two times per week	70 min	Experiment	SF-36	PF, RP, BP, GH, VT, SF, RE, MH, PCS, MCS, T
Luo et al., 2008	40	40	24-form Tai Chi	No exercise	Three times per week	60 min	Experiment	QOL-74	PF, MF, SF, ML
Wang and Wang, 2020	50	50	Doing exercise conducted at least three times per week	Doing exercise conducted less than three times per week	More than three times per week	–	Experiment	QOL	PF, MH, SF, ML, MS, GS, MF
Peng, 2009	65	135	Health-preserving physical exercise	No exercise	–	–	Survey	SF-36	PF, RP, BP, VT, SF, RE, MH, GH, T
Lee et al., 2010	66	73	Chen-style Tai Chi	Daily activities	Three times per week	60 min	Experiment	SF-12	PCS, MCS

*Doing exercise in the morning: a regular physical exercise type for older adults, who choose to do various types of physical exercise in parks, squares, and other places in the morning. It includes Tai Chi, running, walking, badminton, and table tennis. Sporting activities at leisure time: a healthy and scientific way of life, whereby one obtains physical and mental happiness and eliminates fatigue for the purpose of physical exercise. It includes walking, running, gymnastics, square dancing, aerobics, martial arts, and mountain climbing. Light physical exercise: a form of exercise suitable for older adults, characterized by light exercise load, low technical requirements, and its core concept is multi-occasion, multi-functional exercise at any time, emphasizing relaxation, and pleasure. Liuzijue: a traditional Chinese form of physical exercise, that is, the use of breathing with the silent recitation of the six character sounds to adjust the liver, heart, spleen, lungs, and kidneys. Xiyangcao: based on the basic theories of Chinese traditional medicine, such as the doctrine of internal organs, meridians, and essence and energy, as well as the basic knowledge of anatomy, physiology, and sports medicine. It includes movements, such as flexing and stretching, tilting, turning the waist and spine, and folding the body from side to side, together with a unique breathing style. Baduanjin: a traditional Chinese fitness technique that comprises eight movements. No equipment is required and there is no restriction on space. High-intensity exercise: a form of high-intensity exercise, and it includes jogging, mountain climbing, bicycling, and other high-intensity exercises. New exercise prescription: an exercise prescription suitable for Chinese home-based older adults, which contains preparatory activity, warm-up, strength exercise, stretching, and relaxation. 24-form Tai Chi: a traditional Chinese form of physical exercise, that is, derived from the essence of Yang’s Tai Chi. Health-preserving physical exercise: a traditional Chinese health exercise, and it includes Mulan Shan and other traditional health exercise types. Chen-style Tai Chi: an ancient Chinese exercise that works according to the Chinese Theory of Yin-Yang. It involves a series of graceful dance-like movements, which are performed in a slow, rhythmical, and well-controlled manner. Non-physical exercise: sports other than light physical exercise. Tai Chi: a traditional Chinese form of physical exercise. Square dancing: a rhythmic dance performed spontaneously by Chinese residents in open spaces, such as squares and courtyards, for the purpose of fitness usually accompanied by loud rhythmic music. Lack of exercise: walking, irregular exercise, or no exercise. Daily activities: no specific exercise interventions, life as usual.*

Regarding the sample characteristics, for the frequency of exercise, two articles (11%) reported that participants exercised two times per week, three articles (16%) reported that participants exercised three times per week, one article (5%) reported that participants exercised more than two times per week, four articles (21%) reported that participants exercised more than three times per week, three articles (16%) reported that participants exercised more than four times per week, and one article (5%) reported that participants exercised more than five times per week. Five articles (26%) did not clearly report this information.

For the duration of exercise, one article (5%) reported that participants exercised for 30–40 min per session, six articles (32%) reported that participants exercised for more than 30 min, four articles (21%) reported that participants exercised for 60 min, and one article (5%) reported that participants exercised for 70 min. Seven articles (37%) did not clearly report this information.

Several QOL questionnaires were used as measurement tools: (1) the WHOQOL-100 scale (Du and Fang, 2000) is a universal scale of QOL developed by the WHO, comprising 100 items in total with six dimensions, including PD, MD, ID, SR, ED, and SP, and was used by one article (5%); (2) the WHOQOL-BREF scale (Hoben et al., 2016) is a concise scale based on the WHOQOL-100 scale, comprising 26 items in total, including PD, MD, SD, and the ED, and was used in three articles (16%); (3) the SF-36 (Ware and Sherbourne, 1992) is a health questionnaire developed by the Boston Institute of Health in 1990, comprising 36 items in total across nine dimensions, including PF, RP, BP, GH, VT, SF, RE, MH, and health transformation (HT). No points are awarded for the HT dimension, and the remaining dimensions are used to provide two comprehensive indicators: PCS (PF, RP, BP, and GH) and MCS (VT, SF, RE, and MH). In total, 12 articles (63%) used this scale; (4) the Chinese version of the SF-12 (Ware et al., 1996) health questionnaire is used to measure the physiological and psychological components of health-related QOL and contains items representative of each of the eight components of the SF-36. The advantage is that the SF-12 is shorter, yet retains the psychometric measurement characteristics of the SF-36. One study (5%) used this measure; (5) the QOL-74 (Li and Yang, 1995) is a self-assessment questionnaire based on extensive reference to domestic and international quality-of-life questionnaires, comprising 74 items in total, including PF, MF, SF, and ML, and was used in one study (5%); and (6) the QOL scale (Wang and Wang, 2020) assesses the QOL of older adults, including PF, MH, SF, ML, MS, GS, and MF, and was used in one study (5%).

### The Effect of Physical Exercise on the Quality of Life of Healthy Older Adults in China

The results of all the included 19 articles indicated that physical exercise has varying degrees of positive impact on the QOL of healthy older adults in China. Moreover, older adults can promote the improvement in their QOL as long as they are able to exercise. This study suggested that, to improve QOL, older adults should be actively encouraged to participate in various physical exercises.

In terms of types of physical exercise, of the 19 articles, 6 (32%) reported on the effects of comprehensive physical exercise types. Of these six studies, four investigated the effects of body–mind exercise (this is a complementary and alternative therapy that combines body movement with mental concentration. It regards the body, spirit, and external environment as a whole and promotes dynamic balance between the body, mind, and external environment through asanas, pranayama, relaxation techniques, and meditation to maintain health. It contains Liuzijue, Baduanjin, 24-form Tai Chi, and Chen-style Tai Chi). All reported positive effects on mental and physical health-related QOL; the remaining two articles investigated the effects of aerobic exercise on QOL. A new exercise prescription had positive effects on both physical and MH, whereas Xiyangcao positively affected physical health but not MH. Thus, the results indicated that to improve the physical and MH of older adults, physical exercise types that involve body–mind exercises are recommended. The remaining 13 articles (68%) reported regular physical exercise types: doing exercises in the morning, sporting activities at leisure time, light physical exercise, doing a wide variety of exercises, doing exercises conducted at least three times per week, high-intensity exercise, and health-preserving physical exercise. Two articles reported that doing exercises in the morning had positive effects on both physical health and MH; two articles reported that sporting activities at leisure time had positive effects on both physical health and MH; one article reported that light physical exercise had positive effects on all dimensions of QOL; and two articles reported that doing a wide variety of exercise had positive effects on different dimensions of physical health and MH. Three articles reported that doing exercise conducted at least three times per week had positive effects on the different dimensions of physical health and MH; one article reported that doing exercise conducted at least three times per week had positive effects on both MH and physical health, except for BP; one article reported that high-intensity exercise had positive effects on both MH and physical health, except for PF; and one article reported that health-preserving physical exercise had positive effects on both MH and physical health. It can be seen that, because of the complex of physical exercises and numerous types, each physical exercise type has different positive effects on MH and physical health. The analysis indicated that older adults may combine exercises of multiple types to improve their QOL.

In terms of the study design on physical exercise, among the 19 articles included, 12 (63%) were investigative studies and 7 (37%) were experimental studies. In the investigative studies, two articles compared exercise with no exercise, and doing exercises in the morning, doing a wide variety of exercises, sporting activities at leisure time, and doing exercise conducted at least three times per week were more found to be effective in improving the QOL of older adults. One article reported that health-preserving physical exercise was effective in improving the QOL of older adults compared with no exercise. In addition, one article reported that compared with non-light physical exercise, doing exercise conducted less than three times per week, insufficient exercise (walking or no exercise), light physical exercise, doing exercise conducted at least three times per week, and high-intensity exercise were more effective in improving the QOL of older adults. Thus, regardless of the type of exercises compared, the exercise group of the investigative studies exhibited positive effects on QOL. Among the experimental studies, five articles used an active control group (e.g., slow walking, walking, Tai Chi, doing exercise conducted less than three times per week through sporting activities at leisure time, and daily activities), and two studies used a blank control group (i.e., no exercise). Compared with slow walking, Xiyangcao was more effective in improving the QOL of older adults. Compared with walking, Baduanjin was found to be more effective, and compared with Tai Chi, square dancing and a new exercise prescription were more effective. Compared with doing exercise conducted less than three times per week through leisure activities, doing exercise conducted at least three times per week was more effective, and compared with daily activities, Chen-style Tai Chi was more effective in improving QOL. In addition, compared with no exercise, Liuzijue and 24-form Tai Chi improve the QOL of older adults. Thus, regardless of the type of control group used, the exercise group in the experimental studies exhibited positive effects on QOL. Moreover, these studies demonstrated that both active and blank can be used as a control group for studies on QOL in older adults. Investigative studies tended to focus on regular physical exercise types, whereas experimental studies tended to focus on comprehensive physical exercise types.

## Discussion

The literature included in this study showed a steady increase in the number of publications with time, which indicated that the QOL of older adults continues to be of significant interest to scientists (Man et al., 2017). Most studies were investigative studies. In terms of the frequency and duration of exercise, our results showed that exercising for at least 30 min two times per week improves the QOL of Chinese healthy older adults. In terms of measurement tools, numerous scales were used to assess the QOL of healthy older adults in China, of which the SF-36 was the most widely used. The WHOQOL-OLD has not yet been used; it has only been applied to a non-healthy older adult population in China.

Methodological quality evaluation revealed that all included articles were of medium or good quality although several shortcomings remain: (1) some studies did not provide details or specific explanations of the inclusion criteria for subjects; (2) most studies failed to provide baseline values for intervention and control groups, which made it impossible to determine whether baseline levels were similar across groups; (3) most studies did not report blinding of subjects, therapists, and assessors; (4) none of the studies provided point estimates or measures of variability for key outcomes; and (5) the measurement indicators used in some studies were unclear (e.g., exercise frequency, exercise duration, and main research results). Therefore, we encourage future studies to report relevant research information in as much detail as possible, implement blinding, ensure scientific rigor, control bias, and improve the overall quality of the research methodology.

We found that physical exercise has varying degrees of positive effects on the QOL of older adults in China. Regardless of the type of exercise, as long as individuals engaged in some form of exercise, the QOL of older adults could be improved. Previous meta-analyses have reported that exercise improves the cognition, emotion, and sleep quality of older adults (Wu et al., 2015; Kazeminia et al., 2020; Ye et al., 2020), which is conducive to the improvement of the QOL of older adults.

The effect of physical exercise on the QOL of healthy Chinese older adults was mainly reflected in MH and physical health. Different types of exercise had varying effects on the MH and physical health of older adults. For example, in the case of comprehensive physical exercise types, body–mind exercise had a positive impact on the MH and physical health of older adults. When the older adults practiced Liuzijue, Baduanjin, 24-form Tai Chi, and Chen-style Tai Chi, they paid attention to “mind calmness and concentration,” eliminated distracting thoughts, focused their minds, and trained their brains. In addition, the abdominal breathing method also helped with mood regulation and stress reduction. During exercise, every part of the body needs to coordinate to fully mobilize all the senses. Exercise allows older adults to give some direction to their attention; it also cultivates the coordination of the brain and promotes better attention processes. In addition, long-term exercise has a mild effect on the immune function of older adults (Shen et al., 2013), whereas Xiyangcao has been shown to have a positive effect on physical health. In terms of regular physical exercise types, doing exercises in the morning was shown to have a positive effect on the mental state, psychology, and cardiopulmonary function of older adults and effectively improve their physical health and MH of older adults (Zhang et al., 2016). Furthermore, doing exercise conducted at least three times per week had a beneficial effect not only on disease prevention and control, but also on MH. Therefore, we suggest that future studies should focus on specific psychological or physiological aspects of one or more exercise types, and explore the relationship between physical exercise and QOL in older adults.

Different study designs had an impact on the effect of exercise on the QOL of older adults in China. In the investigative studies, irrespective of the type of exercise, the exercise group exhibited a positive effect on QOL. In the experimental studies, most studies used an active control group (i.e., a different form of exercise than the intervention group, such as less exercise or another form of exercise), and results were consistent with those of the studies that used a blank control group. Importantly, both designs showed that exercise was effective. Either type of control group may be used, to compare with the intervention group to explore the effect of physical exercise on QOL in older adults. To better explain the results, we suggest including two control groups in the future; namely, a non-exercise group and a group which exercise not used in the intervention. Moreover, to improve the values of the studies, we suggest conducting studies on multiple forms of exercise, regardless of whether the study used an investigative or experimental design. In addition, because of the small number of experimental studies, we encourage additional experimental studies to clarify the specific impact of physical exercise on QOL in older adults, the differences between the different forms of physical exercise, and the optimal dose of physical exercise. Such studies will provide a basis for the formulation of specific physical exercise programs to improve the QOL of older adults in China.

In this study, two reviewers independently carried out literature screening, data extraction, and quality evaluation, and a third reviewer was involved in a discussion to ensure the quality of the systematic review. However, several limitations must be noted. Firstly, the literature included in this study lacked relevant information, such as the results of various dimensions and exercise frequency. Secondly, because of the limited data quality of some scales, it was not possible to include all the results of the QOL scales in the analysis. Finally, we included only healthy older adults in China, and future studies may consider other groups to enrich our findings.

## Conclusion

Our systematic review showed that physical exercise has a positive effect on the QOL of healthy older adults in China. Body–mind exercises are effective in improving the physical health and MH of older adults, whereas Xiyangcao only had a positive effect on physical health. However, given that the methodological quality of the literature was generally low, we recommend that relevant training is provided to researchers to improve the quality of research. Future studies should report relevant study information in as much detail as possible and use consistent research methods to obtain more reliable results.

## Data Availability Statement

The original contributions presented in the study are included in the article/supplementary material, further inquiries can be directed to the corresponding author.

## Author Contributions

LW, YH, and LZ: conceptualization. LW, YT, and RH: methodology and formal analysis. LW and YT: software. LW: writing—original draft preparation. LZ: writing—review and editing, and supervision. RH: visualization. All authors have read and approved this manuscript to submit for publication.

## Conflict of Interest

The authors declare that the research was conducted in the absence of any commercial or financial relationships that could be construed as a potential conflict of interest.

## Publisher’s Note

All claims expressed in this article are solely those of the authors and do not necessarily represent those of their affiliated organizations, or those of the publisher, the editors and the reviewers. Any product that may be evaluated in this article, or claim that may be made by its manufacturer, is not guaranteed or endorsed by the publisher.

## Abbreviations

PF: physical function
RP: role-physical
BP: bodily pain
GH: general health
VT: vitality
SF: social function
RE: role-emotional
MH: mental health
PCS: physical component summary
MCS: mental component summary
ML: material life
MS: mental state
GS: general state
MF: mental function
PD: physiological domain
MD: mental domain
SD: social domain
ED: environmental domain
ID: independent domain
SP: spiritual pillar
SR: social relations.
